# Predictive value of lymphocyte-to-monocyte ratio (LMR) and neutrophil-to-lymphocyte ratio (NLR) in patients with oesophageal cancer undergoing concurrent chemoradiotherapy

**DOI:** 10.1186/s12885-019-6157-4

**Published:** 2019-10-26

**Authors:** Ke-Jie Li, Xiao-Fang Xia, Meng Su, Hui Zhang, Wen-Hao Chen, Chang-Lin Zou

**Affiliations:** 1 Wenzhou Medical University, Wenzhou, 325000 People’s Republic of China; 2 Department of Radiotherapy and Chemotherapy, The First Affiliated Hospital of Wenzhou Medical University, WenZhou, China

**Keywords:** Oesophageal cancer, Blood inflammatory markers, Concurrent chemoradiotherapy, Overall survival

## Abstract

**Background and objectives:**

The survival rate of patients with advanced oesophageal cancer is very low and can vary significantly, even among patients with the same TNM stage. It is important to look for indicators that are economical and readily available to predict overall survival. The aim of this study was to determine whether lymphocyte-to-monocyte ratio (LMR) and neutrophil-to-lymphocyte ratio (NLR) could be potential predictors of survival in patients with advanced oesophageal squamous cell carcinoma (ESCC) undergoing concurrent chemoradiotherapy.

**Methods:**

Differences in survival among 204 patients with advanced oesophageal cancer who underwent concurrent chemoradiotherapy were collected and analysed. Univariate and multivariate COX regression analyses were used to investigate the association between blood inflammatory markers and patient survival before treatment.

**Results:**

Univariate COX regression analyses showed that a history of alcohol use, neutrophil count, LMR, NLR, tumour length, and N stage were significantly associated with the survival of tumour patients receiving concurrent chemoradiotherapy. Multivariate COX regression analysis showed that NLR and LMR were predictors of outcome in tumour patients receiving chemoradiotherapy. According to receiver operating characteristic (ROC) curve analysis, the AUC of LMR and NLR was 0.734 and 0.749, and the best cutoff point for LMR and NLR was 3.03 and 2.64, respectively.

**Conclusions:**

LMR and NLR can be used to predict the survival of patients with advanced oesophageal cancer receiving concurrent chemoradiotherapy, thereby providing clinicians with suggestions for further treatment options.

## Background

Oesophageal cancer is one of the most common tumours in the world. According to the latest statistics, the incidence of oesophageal cancer ranks fourth among all tumours [1]. There are two main pathological types of oesophageal cancer: oesophageal squamous cell carcinoma (ESCC) and oesophageal adenocarcinoma (EAC), and ESCC is the more common type, especially in Asia and Africa [2]. Due to the lack of specific symptoms in the early stage of oesophageal squamous cell carcinoma, most patients are in the advanced stage at the time of diagnosis and lose the opportunity for surgery. In China, approximately 38,000 people die of oesophageal cancer each year [3].

For patients with inoperable oesophageal cancer, there are a variety of treatments, including radiotherapy, chemotherapy, concurrent chemoradiotherapy, targeted therapy and immunotherapy [4]. In clinical work, concurrent chemoradiotherapy is the main means we use to improve the survival of patients with advanced oesophageal cancer. Radiation therapy has played an increasingly important role in the treatment of oesophageal cancer [5]. With the continuous updating and development of technology, techniques such as intensity-modulated radiation therapy (IMRT), volumetric arc therapy (VMAT) and proton therapy have been used to treat patients with advanced oesophageal cancer. Studies by Stefania Martini and Francesca Arcadipane et al. have demonstrated that volumetric modulated arc therapy (VMAT) is an effective and safe strategy for treatment of patients with advanced oesophageal cancer [6–8]. According to a study by Samantha Warren et al., proton therapy for patients with advanced oesophageal cancer may have lower blood toxicity [9]. Similarly, a retrospective analysis by Mian Xi and Cai Xu et al. showed that the metrological advantages of proton therapy play an important role in improving the overall survival of patients with advanced oesophageal cancer [10]. These methods can not only improve the therapeutic effect on the tumour area but also better protect normal tissues, such as the heart and lungs. However, the therapeutic effects of these treatments in individuals with oesophageal cancer remain uncertain. Because each individual patient is different, even if their clinical TNM staging is the same, their survival can vary greatly after receiving similar treatment. We have observed that some stage IV patients can continue to survive for several years, and some patients die within a few months after diagnosis. We expect a positive treatment response for patients who expect to live longer and seek prolong their survival as much as possible. For patients who are expected to have a shorter survival time, some palliative treatments can be used to alleviate suffering and avoid the side effects and financial burden caused by overly aggressive treatment. Recently, some studies have shown that certain protein markers or genes can predict the survival of patients with oesophageal cancer [11, 12], but the acquisition of these indicators is very cumbersome, imposes a large economic burden and requires an extended wait time for patients. Therefore, it is especially important to identify blood inflammatory markers that are easy to obtain and can predict patient survival.

Currently, inflammatory markers such as LMR and NLR are widely studied in predicting the prognosis of oesophageal cancer. Based on their research, Dawei Yuan and colleagues believe that NLR has great value in predicting the disease-free survival and overall survival of patients with oesophageal cancer after surgery [13]. Noriyuki Hirahara and his team used a scoring system based on LMR, NLR and PLR and reported that LMR and NLR are effective predictors of overall survival in patients with oesophageal cancer [14]. Conway AM and Salih Z et al. concluded that NLR is an independent prognostic factor for oesophageal cancer and can be used in conjunction with AJCC8 clinical staging to predict baseline prognosis stratification in patients newly diagnosed with resectable, oesophageal adenocarcinoma [15]. The above studies suggest that LMR and NLR are potential clinical biomarkers that are easy to calculate, can be repeatably obtained and have a low cost. However, there have been no reports on the correlation between inflammation-related indicators and the overall survival of oesophageal cancer patients after they receive radiotherapy and chemotherapy. The aim of our study was to determine whether LMR and NLR could be predictors of survival in patients with advanced oesophageal cancer that receive concurrent chemoradiotherapy.

## Materials and methods

### Patient selection

The study included 204 patients with advanced oesophageal cancer treated at the First Affiliated Hospital of Wenzhou Medical University between 2010 to 2014. The patients were diagnosed with advanced oesophageal cancer at the time of diagnosis and thus had lost the best opportunity for surgery. The inclusion conditions were as follows: (1) patients between 18 and 85 years of age; (2) pathologically confirmed oesophageal squamous cell carcinoma; (3) only received concurrent chemoradiotherapy after diagnosis; (4) exhibited no significant adverse effect on blood inflammation diseases, such as vasculitis and systemic lupus erythaematosus. We collected blood inflammatory markers from these patients prior to treatment, including white blood cells, neutrophils, monocytes, lymphocytes, LMR, NLR, NMR, and CEA. In addition, some basic characteristics of the cancer patients, such as age, gender, smoking history, drinking history, and ECOG score, were recorded, as well as tumour features, including pathological type, degree of differentiation, lymph node metastasis and tumour location, length, and width, which were obtained from CT, endoscopic ultrasound, and pathological diagnosis.

### Treatment protocol

All patients enrolled received concurrent chemoradiotherapy. A total dose of up to 54 Gy was delivered via standard fractionated radiotherapy in 30 fractions (on work days; 1.8 Gy per fraction; over a 6-week cycle). The principle of target mapping is as follows: GTV represents the primary oesophageal lesion and shows the length of the tumour as indicated by oesophageal angiography and/or oesophagoscopy and/or intraluminal ultrasound. The length of the primary tumour is combined with CT and PET/CT imaging results and the scope of invasion. An enlarged metastatic lymph node is represented by GTVnd: a positive lymph node is defined as a lymph node with the largest short diameter ≥ 1 cm detected by CT/MRI or a node with a size not more than 1 cm but showing obvious necrosis and ring enhancement. CTV includes GTV and the GTVnd+ lymph node drainage area and an additional 0.8 cm outside GTV and GTVnd (plane), 3–5 cm in GTV and GTVnd (up and down), or 1.5 on the CT level with lymph node metastasis − 2.0 cm. The PTV is 0.5 cm on the basis of CTV. The concurrent chemotherapy regimen was paclitaxel 135 mg/m2 combined with cisplatin 25 mg/m2 once every 3 weeks per cycle. The study was approved by the Ethics Committee of the First Hospital affiliated with Wenzhou Medical University. Because all the patients in this retrospective study had died, informed consent was obtained from their family members or their pretreatment authorized recipients.

### Evaluation strategy

The primary end point of assessment was patient overall survival (OS), which was defined as the time from randomization to the time of death from any cause. For subjects who had missed their follow-up visits prior to death, their last follow-up was counted as the time of death. Secondary assessment endpoints were progression-free survival (PFS) and objective response rate (ORR). PFS was defined as the time between the onset of randomization and the progression (or any aspect) of tumourigenesis or death (for any reason). The ORR was defined as the proportion of patients whose tumour volume was reduced to a predetermined value and was maintained for the minimum time limit, which was the sum of complete and partial relief. In other words, ORR = CR + PR. CR indicates that the tumour completely disappeared for more than 1 month; PR indicates that the sum of the largest diameters of the tumour was reduced by at least 30% and was maintained for at least 4 weeks.

### Data statistics

All statistical analyses were performed using a social science statistical software package, version 22.0 (SPSS Inc., Chicago, IL, USA). A receiver operating characteristic curve was used to select the best cutoff value for blood inflammatory indicators and to stratify the indicators. The Kaplan-Meier method was used to plot survival curves. A chi-square test was used to analyse correlations between predictors and tumour parameters. The routine parameters included haematology markers (white blood cells, neutrophils, monocytes, lymphocytes, LMR, NLR, NMR, PLR, and CEA) and clinical pathology characteristics (sex, age, drinking history, tumour site, tumour stage, and ECOG score). Univariate analysis was performed to determine which variables were associated with the survival of the tumour patient. Multivariate COX regression analysis was used to identify predictors of advanced oesophageal cancer. *p* < 0.05 was considered statistically significant.

## Result

A total of 204 patients with oesophageal cancer were included in this study. The age distribution of the patients was between 38 and 85 years, with a median age of 65.8 years; other specific clinical and pathological features of the patients are shown in Table 1.

**Table 1:** Basic physiological and physiological characteristics of 204 patients

Characteristic	No of people(%)
No of people(%)	204
Sex	
Female	33 (16.2%)
Male	171 (83.8%)
Age	
Median	65.8 years
Range	38–85
65 years old or older	102 (50%)
Under 65 years old	102 (50%)
History of smoking	
Yes	104 (50.9%)
No	100 (49.1%)
Drinking history	
Yes	100 (49.1%)
No	104 (50.9%)
Differentiation	
Highly differentiation	51 (25%)
Medium differentiation	96 (47.1%)
Low differentiation	57 (27.9%)
Tumor site	
Upper thoracic portion	102 (50%)
Middle thoracic portion	78 (38.2%)
Low thoracic portion	24 (11.8%)
Tumor length (cm)	
Median	4.8 cm
Range	0.9–11.3 cm
More than 4 cm	104 (50.9%)
Less than 4 cm	100 (49.1%)
T-staging	
T1 + T2	71 (34.8%)
T3 + T4	133 (65.2%)
N-staging	
N0	75 (36.8%)
N1 + N2	129 (63.2%)
ECOG score	
0 point	174 (85.3%)
1 point	21 (10.3%)
2 point	9 (4.4%)

The median follow-up time was 11.5 months (range: 2.1 to 77.4 months). According to univariate COX regression (Table 2), drinking history, tumour length, neutrophils, NLR, and LMR were associated with survival in patients undergoing concurrent chemoradiotherapy for oesophageal cancer.

**Table 2:** Univariate COX regression analysis of the relationship between pathophysiological parameters and survival time of patients

Parameter	OR	95% CI	*P*
Sex	1.121	0.770–1.631	0.551
Age	1.119	0.848–1.476	0.427
Smoking history	1.141	0.864–1.506	0.352
Drinking history	1.333	1.005–1.769	0.046
Differentiation	0.972	0.715–1.321	0.856
Tumor site	0.858	0.647–1.137	0.286
Tumor length	1.365	1.033–1.802	0.028
Tumor width	1.154	0.876–1.520	0.309
T-staging	1.468	1.095–1.969	< 0.05
N-staging	1.707	1.271–2.294	< 0.05
ECOG score	1.173	0.974–1.731	0.423
Lymphocytes	0.896	0.677–1.186	0.443
Neutrophils	1.545	1.162–2.053	0.003
Monocytes	1.176	0.891–1.574	0.252
LMR	0.278	0.205–0.376	< 0.05
NLR	2.223	1.670–2.960	< 0.05
CEA	0.916	0.666–1.260	0.590
NMR	0.900	0.591–1.370	0.622

Multivariate COX regression (Table 3) analysis was performed with the statistically significant indicators in the univariate COX regression analysis, and the results suggested that NLR (OR 2.233 (95% CI 1.67–2.96), *p* < 0.005), LMR (OR 0.278 (95% CI 0.205–0.376), *p* < 0.05), and T stage are predictors of survival in patients with oesophageal cancer. Patients with high LMR values showed longer survival than patients with low LMR values, whereas patients with a lower NLR had a longer survival period than patients with high NLR values.

**Table 3:** Multivariate COX regression analysis of the relationship between clinical variables and patient survival

Parameter	OR	95% CI	*P*
Drinking history	1.103	0.813–1.497	0.528
Tumor length	0.880	0.611–1.268	0.492
T-staging	1.598	1.037–2.462	0.034
N-staging	0.957	0.687–1.333	0.957
Neutrophils	1.197	0.880–1.628	0.253
LMR	0.331	0.238–0.459	< 0.05
NLR	1.597	1.151–2.215	< 0.05

**Fig. 1:**
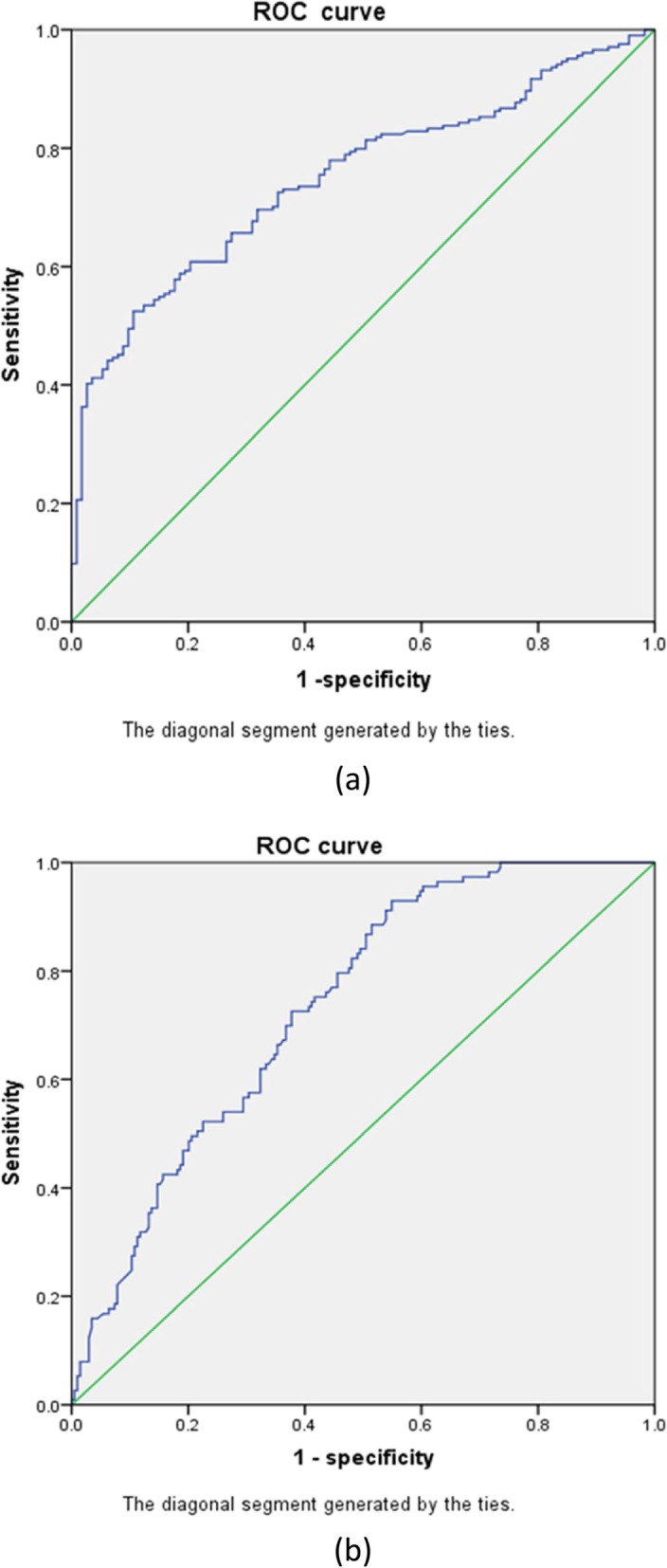
Receiver operating characteristic (ROC) curve plotted to determine the value of a statistically significant variable in the COX regression model for NLR (**a**) and LMR (**b**). According to ROC analysis, the area under the curve of NLR and LMR was 0.749 and 0.734, respectively, and the optimal cutoff point was 2.64 and 3.03, respectively

A receiver operating characteristic (ROC) curve (Fig. 1) was plotted to assess the value of statistically significant variables in the COX regression model for NLR and LMR. The area under the curve of LMR and NLR was 0.734 and 0.749, and the optimal cutoff value of LMR and NLR was 3.03 and 2.64, respectively. The ROC curve is shown in Fig. 1.

**Fig. 2:**
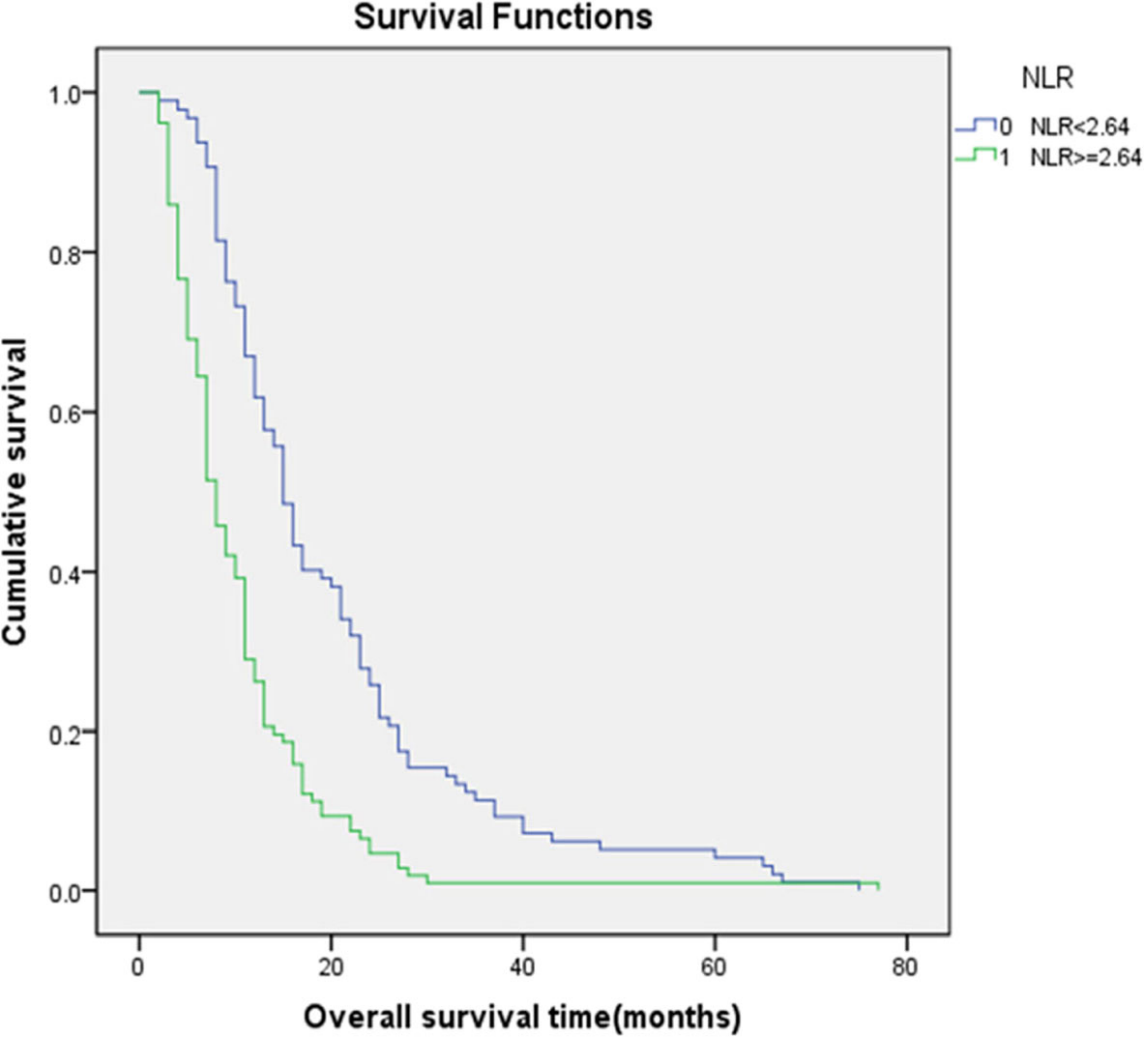
Kaplan-Meier survival curves for patients with advanced oesophageal cancer in different NLR groups. The blue curve represents the overall survival of patients with an NLR less than 2.64, while the green curve represents the overall survival of patients with an NLR greater than or equal to 2.64. The mean survival time of patients in the low NLR group was 19.8 months, and the mean survival time of patients in the high NLR group was 10.3 months, with a *p* < 0.05, indicating a significant difference between the two groups

**Fig. 3:**
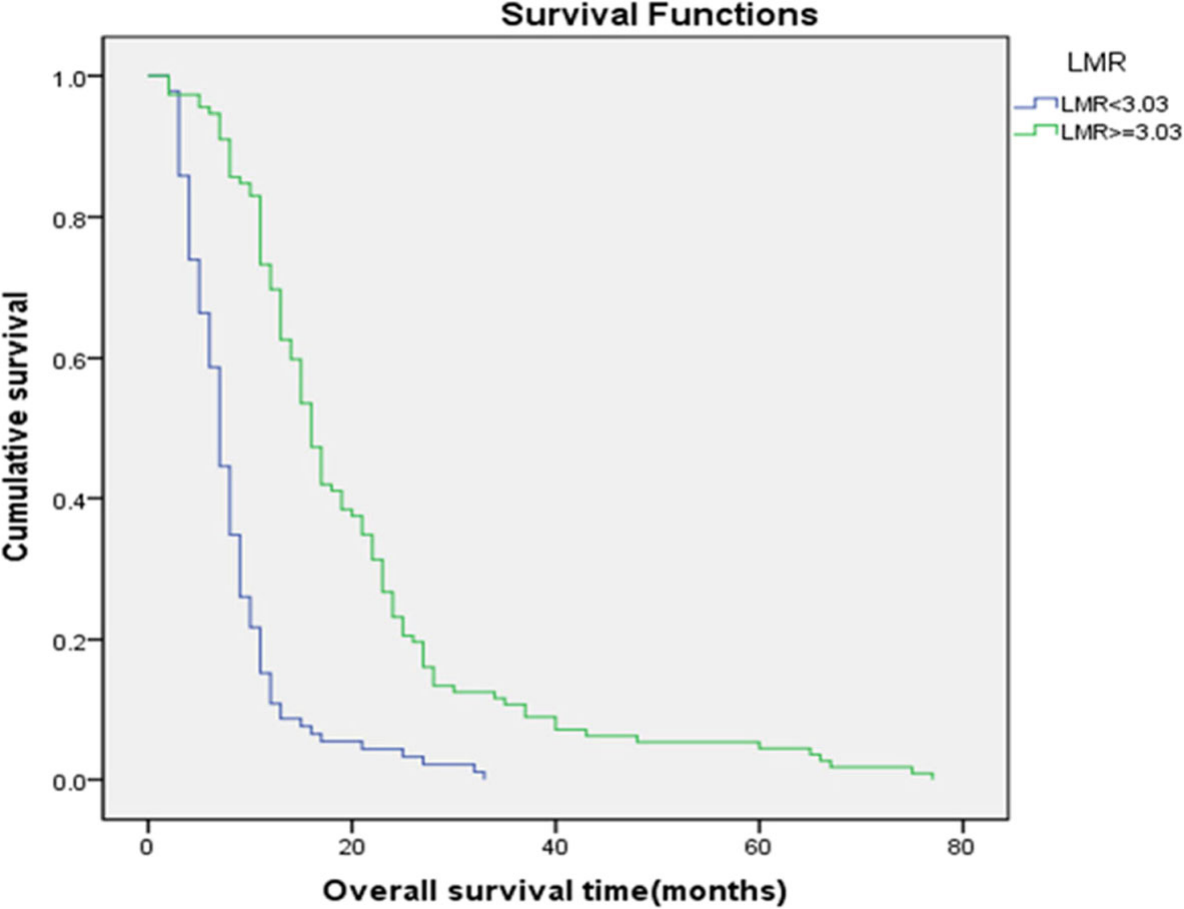
Kaplan-Meier survival curves for patients with advanced oesophageal cancer in different LMR groups. The blue curve represents the overall survival of patients with an LMR less than 3.03, while the green curve represents the overall survival of patients with an LMR greater than or equal to 3.03. The mean survival time of patients in the group with an LMR less than 3.03 was 8.3 months, while that of patients in the group with an LMR greater than or equal to 3.03 was 20.2 months, with a *p* value less than 0.05, indicating a significant difference between the two groups

Kaplan-Meier analysis was applied to construct a survival curve. In patients with an NLR (Fig. 2) less than 2.64, the mean survival was 19.8 months, and the median survival was 15 months. In patients with an NLR greater than or equal to 2.64, the mean survival was 10.3 months, and the median survival was 8 months. In patients with an LMR (Fig. 3) less than 3.03, the mean survival was 8.3 months, and the median survival was 7 months. In patients with an LMR greater than or equal to 3.03, the mean survival was 20.2 months, and the median survival was 16 months. Both *p* values were less than 0.05, indicating statistical significance.

Chi-square tests were used to analyse the relationship between LMR and NLR and conventional tumour parameters. Analyses showed that NLR (Table 4) was associated with N stage (*p* < 0.05), tumour location (*p* = 0.017), tumour stage (p < 0.05), and treatment efficacy (p < 0.05), while LMR (Table 5) was associated with efficacy of treatment (*p* < 0.05). At the same time, multivariate logistic regression showed that NLR (OR 1.918 (95% CI 1.406–2.617) *p* < 0.05), and LMR (OR 0.337 (95% CI 0.245–0.463) *p* < 0.05) were significantly associated with PFS.

**Table 4:** Association of pathological features and NLR in patients

Characteristic,*n* = 204	NLR < 2.64	NLR > =2.64	*P*
N-staging			
N0	26	49	
NI + N2	71	58	< 0.05
Treatment efficacy			
CR + PR	63	34	
SD + PD	34	73	< 0.05
T-staging			
T1 + T2	38	33	
T3 + T4	59	74	0.212
Tumor location			
Upper thoracic	40	62	
Lower thoracic	57	45	0.017
Age			
< 65 years old	48	54	
> =65 years old	49	53	0.889
Sex			
Male	79	92	
Female	18	15	0.066
Drinking history			
Yes	41	59	
No	56	48	0.379
Differentiation			
Medium-high differentiation	71	76	
Low differentiation	26	31	0.73

**Table 5:** Association of pathological features and LMR in patients

Characteristic,n = 204	LMR < 3.03	LMR > =3.03	*P*
Sex			
Male	77	94	
Female	15	18	0.964
Age			
< 65 years old	49	53	
> =65 years old	43	59	0.399
N-staging			
N0	39	36	
NI + N2	53	76	0.131
Treatment efficacy			
CR + PR	26	71	
SD + PD	66	41	< 0.05
Drinking history			
Yes	42	58	
NO	50	54	0.402
Differentiation			
Medium-high differentiation	65	82	
Low differentiation	27	30	0.685

## Discussion

Oesophageal cancer is a malignant tumour with high incidence. Its occurrence and development are related to age, gender, occupation, race, region, living environment, eating habits, and genetic susceptibility, among other factors. Long-term drinking of strong alcohol, habitual smoking, eating food that is too hard, overheating, eating too fast can cause irritation, and chronic inflammation can lead to oesophageal cancer. Despite the continuous innovation in surgical methods, improvement in chemotherapy regimens, adjustment of radiotherapy plans, and continuous development and marketing of targeted therapeutics and immunotherapeutics, the survival rate of patients with advanced oesophageal cancer is still very low. Moreover, patients with oesophageal cancer with the same TMN stage have widely varying survival outcomes after receiving similar treatment, and some patients even rapidly deteriorate after treatment with standardized chemotherapy. Therefore, predicting a patient’s likely survival before treatment can help the clinician determine prognosis and provide individualized treatment. Currently, clinical TNM staging is considered the gold standard for predicting outcomes and determining treatment options. However, because accurate TNM staging requires post-operative pathology, it is difficult for TNM staging to predict survival and determine further treatment strategies for patients with advanced oesophageal cancer who are inoperable. In view of the above facts, there is an urgent need to explore prognostic biomarkers that are easily evaluated and reproducible for patients with advanced inoperable oesophageal cancer.

Among the many potential biomarkers, such as genetic, immunological, and haematological markers, systemic inflammatory markers in peripheral blood are receiving increasing attention for prediction of tumour recurrence, metastasis, and prognosis [16–18]. Recent studies have shown that absolute inflammatory cell counts in peripheral blood (neutrophils, white blood cells, lymphocytes and monocytes) and ratios based on these cell counts (NLR, PLR and LMR) may play a key role in predicting the overall survival of patients with tumours, including colorectal cancer [19], head and neck cancer [20], non-small cell lung cancer [21]. Chen and his colleagues have shown that red cell distribution width (RDW) has predictive value for determining the survival of patients with oesophageal cancer [22]. Yusuke Ishibashi and his team believe that CAR is the most important predictor of OS in patients with oesophageal cancer [23]. A study by Hongdian Zhang and Xiaobin Shang et al. showed that systemic immune-inflammation index (SII) and prognostic nutritional index (PNI) are powerful indicators of invasive biology and poor prognosis in ESCC patients. The combination of SII and PNI can improve the accuracy of prognosis in patients with oesophageal cancer [24]. A retrospective analysis by Apostolos Gaitanidis and his colleagues showed that markers of the systemic inflammatory response are prognostic factors in patients with pancreatic neuroendocrine tumours [25]. Some previous studies have shown that inflammatory markers may affect the survival of cancer patients in many ways. Cancer-associated inflammation alters and polarizes the tumour microenvironment, and although it is not associated with tumour necrosis, it can increase the propensity for tumour recurrence and metastasis [26, 27]. Inflammatory cells can not only inhibit the proliferation and migration of tumour cells [28], but also eliminate residual tumour cells and micrometastases [29]. Lymphocytes play an important role in promoting anti-tumour immunity, and lymphopenia may impair the efficacy of the immune system. For example, if the level of effector T cells is insufficient, cell-mediated cytotoxicity may be weakened, and the killing effect on tumour cells is also weakened [30].

In our study, it was demonstrated that LMR and NLR may be predictors of overall survival in patients with advanced oesophageal cancer after administration of chemoradiotherapy. In this study, we analysed the relationship between LMR and NLR and survival in patients with advanced oesophageal cancer undergoing chemoradiotherapy. First, we used a receiver operating characteristic curve (ROC) to analyse the optimal cutoff value for predicting the OS index, and the optimal cutoff value for LMR and NLR was 3.03 and 2.64, respectively, according to the ROC curve. Univariate and multivariate logistic regression analyses were then used to show that NLR (OR 2.233 (95% CI 1.67–2.96), *p* < 0.05) and LMR (OR 0.278 (95% CI 0.205–0.376), p < 0.05) are closely related to the survival of patients with advanced oesophageal cancer receiving chemoradiotherapy. The analysis showed that the overall survival of patients with oesophageal cancer with an LMR less than 3.03 before treatment was significantly shorter than that of those with an LMR greater than 3.03. Moreover, the value of LMR before treatment was positively correlated with the survival of patients with oesophageal cancer. The value of NLR before treatment was negatively correlated with the survival of patients with oesophageal cancer, because the average survival time of patients with oesophageal cancer with an NLR greater than 2.64 and an NLR less than 2.64 was 10.3 months and 19.8 months, respectively. When we used the patient progression-free survival time as the secondary end point, we drew the same conclusion. At the same time, we performed a chi-square test analysis and found that LMR is related to clinical stage and treatment effect and that NLR is related to tumour location, N stage, clinical stage and treatment effect.

Although our data and results were accurately refined and calculated, this study has some limitations. First, only 204 patients were examined in this study, which may result in unstable results due to the small sample size. Second, TNM staging is only clinical staging, and although we used ultrasound endoscopy and contrast-enhanced CT and PET/CT to determine staging, these methods may limit the ability to assess the prognosis of ESCC compared with pathological staging. Furthermore, our study only verifies the predictive value of LMR and NLR for patient survival, and we need to establish a systematic mathematical prediction model to serve the clinic. Finally, this was a single-centre and retrospective study; thus, all the included patients were from a single hospital, and the conclusions were not verified in other centres. Therefore, this study requires further prospective trials at multiple centres to confirm the reproducibility of the results in heterogeneous populations.

## Conclusion

LMR and NLR are predictors of outcome for patients with advanced oesophageal cancer who receive concurrent chemoradiotherapy. The value of LMR and NLR can help clinicians predict the survival of patients and to select appropriate treatment schemes.

## Data Availability

The dataset in this study is available by request from the corresponding author. For more information, please contact the corresponding author.

## Abbreviations

AUC: Area Under The Curve
CI: Confidence Interval
CR: Complete Remission
CT: Computed Tomography
CTV: Clinical Target Volume
ESCC: Oesophageal Squamous Cell Carcinoma
GTV: Gross Target Volume
LMR: Lymphocyte-To-Monocyte Ratio
MRI: Magnetic Resonance Imaging
NLR: Neutrophil-To-Lymphocyte Ratio
ORR: Objective Response Rate
OS: Overall Survival
PET-CT: Positron Emission Tomography-Computed Tomography
PFS: Progression-Free Survival
PR: Partial Remission
PTV: Planning Target Volume
ROC: Receiver-Operating Characteristic
